# Choroidal Thickness in Indigenous Australian Children

**DOI:** 10.1167/tvst.9.12.28

**Published:** 2020-11-20

**Authors:** Scott A. Read, Rebecca A. Cox, David Alonso-Caneiro, Shelley Hopkins, Joanne M. Wood

**Affiliations:** 1 Queensland University of Technology (QUT), Centre for Vision and Eye Research, School of Optometry and Vision Science, Kelvin Grove, Queensland, Australia

**Keywords:** choroid, childhood, indigenous, refractive error

## Abstract

**Purpose:**

This study aimed to examine the choroidal thickness profiles in visually normal Australian Indigenous children, given the important role of the choroid in refractive error and a range of ocular diseases.

**Methods:**

Choroidal thickness was assessed across the central 5 mm macular region using enhanced depth imaging spectral domain optical coherence tomography, in 250 children enrolled in an elementary school and a secondary school in rural Queensland, Australia. One hundred (40%) of these children identified as Indigenous Australians.

**Results:**

The subfoveal choroid was significantly thicker in Indigenous children (mean 369 ± 75 µm), compared to non-Indigenous children (355 ± 73 µm; *P* = 0.03). Subfoveal choroidal thickness was also significantly associated with age (β = +7.6, r^2^ = 0.105, *P* = 0.003), and axial length (β = −19.9, r^2^ = 0.030, *P* < 0.001). A significantly thicker choroid in Indigenous children was also found in analyses across the central 5 mm macular region (*P* = 0.008). A significant interaction between Indigenous status and meridian was observed (*P* = 0.007) with the largest differences between Indigenous and non-Indigenous children being in the nasal and inferonasal meridians.

**Conclusions:**

This study establishes the normative characteristics of macular choroidal thickness in Indigenous Australian children and demonstrates a significantly thicker choroid compared to non-Indigenous children from the same geographic region. These results may have implications for our understanding of factors predisposing or protecting Australian Indigenous people from a range of conditions associated with choroidal thickness.

**Translational Relevance:**

The significantly thicker choroid in Australian Indigenous children should be considered in clinical diagnoses and management of conditions associated with choroidal changes.

## Introduction

Knowledge of the normal characteristics of the choroidal structure in the human eye is important given the role of the choroid in normal retinal function,^1^ mechanisms regulating eye growth,^2^ and in a range of different ocular diseases.^3^ This has been facilitated by recent advances in optical coherence tomography (OCT) imaging technology,^4^ which has enabled significant improvements in our knowledge regarding the normal in vivo human choroid, and how it changes in the presence of refractive error and disease.

In recent decades, in vivo choroidal changes in the human eye associated with refractive error have been well documented. Cross-sectional studies of both adults^5^^–^^7^ and children^8^^–^^14^ demonstrate that the type and level of refractive error is associated with differences in choroidal thickness, with higher levels of myopia typically associated with a thinner choroid. Longitudinal studies in childhood also demonstrate an association between changes in choroidal thickness and the rate of axial eye growth, with choroidal thinning associated with more rapid axial eye growth, and a thickening of the choroid associated with slower eye growth.^15^^–^^18^

Choroidal structural changes have also been shown to be associated with a range of different ocular diseases.^19^^–^^26^ Increases in choroidal thickness are associated with a range of diseases that can cause vision loss due to the accumulation of sub-retinal fluid in the central retina, such as central serous chorioretinopathy,^19^ polypoidal choroidal vasculopathy,^25^ and diabetic macular edema.^24^ Studies have also demonstrated that the fellow unaffected eyes of patients with unilateral central serous chorioretinopathy exhibit a significantly increased choroidal thickness,^26^ and that first and second degree relatives of patients with central serous chorioretinopathy have thicker than normal choroids.^27^ These findings suggest that a thicker than normal choroid (known as “pachychoroid,” which has been defined by some studies as a choroidal thickness of greater than 395 µm^27^) may be a risk factor for the development of these central retinal conditions. In contrast, other retinal diseases, such as non-exudative AMD, have been found to be associated with a thinner choroid, with longitudinal studies showing that a thinner initial subfoveal choroidal thickness is associated with a greater rate of progression of geographic atrophy.^22^

A range of demographic factors are also known to influence the normal choroidal thickness. In adults, choroidal thickness has been reported to be greater in males than females.^28^^,^^29^ Findings in pediatric populations are less consistent, however, with a number of studies reporting no significant difference in the choroidal thickness of males and females.^8^^–^^10^^,^^30^ In children without substantial refractive error, significant increases in choroidal thickness with increasing age in childhood have been reported,^8^^,^^13^^,^^30^ and it has been suggested that this natural increase in choroidal thickness may function as a buffer against the rapid eye growth that occurs during childhood. In cross-sectional and longitudinal studies of children with a higher prevalence of myopia, significant decreases in choroidal thickness are reported,^10^^,^^14^^,^^18^ consistent with an interaction between the normal developmental changes in the choroid and the changes associated with the development and progression of myopia in childhood. In adults, a thinning of the choroid has been consistently reported to be associated with older age.^7^^,^^29^

Only a small number of studies to date have examined whether ethnicity influences choroidal thickness.^6^^,^^31^^,^^32^ Although some studies report no significant difference in choroidal thickness among white, African, and Asian populations,^31^ other studies report significant effects of ethnicity on choroidal thickness in some regions of the choroid.^6^^,^^32^ In a population of subjects without significant myopia, Bafiq and colleagues^32^ reported that healthy adults of African ethnicity exhibited a thinner choroid than South Asian and white adults in the temporal choroidal region only. Conversely, in an ethnically diverse cohort of myopic young adults, Harb et al.^6^ reported a thicker choroid in African participants compared to Asian and white populations, in the nasal choroidal regions only. Although the small number of studies in this field suggest a potential influence of ethnicity upon choroidal thickness, it is noteworthy that the choroidal characteristics of a number of different populations have yet to be examined in detail (for example Indigenous Australian populations) and, importantly, the relevance of these variations is as yet unknown. Work is therefore needed to further characterize the effects of ethnicity upon the choroid.

In Australia, Aboriginal and/or Torres Strait Islander people comprise approximately 3% of the population.^33^ It is well documented that there is a disparity in the rates of vision impairment and eye disease between Indigenous and non-Indigenous populations in Australia.^34^^,^^35^ The 2016 National Eye Health Survey (NEHS) reported that the prevalence of vision impairment in Indigenous Australians was approximately three times higher than that of non-Indigenous Australians, with the leading causes of vision loss in Indigenous Australians being uncorrected refractive error, cataract, and diabetic retinopathy.^34^ These disparities in vision and eye health outcomes in Australian Indigenous populations, underscore the importance of better understanding the normative ocular characteristics of Indigenous populations.

Although only a small number of studies have provided detailed information regarding refractive error in Australian Indigenous populations, studies of both adults and children report a very low prevalence of myopia compared to other populations.^36^^,^^37^ In a population of 1884 Indigenous Australian adults living in central Australia, Landers and colleagues^37^ documented a myopia prevalence of only 11% in Indigenous Australians. Hopkins et al.,^36^ in a study of Indigenous children aged between 5 and 13, reported a prevalence of myopia of only 1.7%. Given the association between choroidal thickness and myopia, understanding the choroidal characteristics of Indigenous Australian populations may also provide insights into the ocular characteristics that are potentially protective against myopia development.

In this study, we aimed to characterize the normal topographical characteristics of choroidal thickness in Indigenous Australian children. Given the strong association between choroidal thickness with a range of eye diseases and refractive error, an improved understanding of the normal choroidal characteristics of Indigenous children is important for future diagnosis of eye disease and better understanding of eye growth and refractive error in this population.

## Methods

All children enrolled at a primary school (*n* = 242) and a high school (*n* = 347) in rural South East Queensland, Australia, were invited to participate in this research. These schools were selected due to their high enrollment of Indigenous children. Two hundred fifty children aged between 4 and 18 years (mean age 11.7 ± 3.4 years) who agreed to participate in this study and had normal vision and ocular health were included in the analyses presented in this paper. Approval from the Queensland University of Technology human research ethics committee and from the Queensland Government Department of Education was provided before commencement of the study and written parental consent and child assent was provided by all participants. If children were unable to provide written assent, then verbal assent was provided. All children were treated according to the tenets of the Declaration of Helsinki. Indigenous status (children who identify as Aboriginal and/or Torres Strait Islander) was established based upon a questionnaire completed by each child's parent or guardian. Both Indigenous and non-Indigenous children were enrolled in the study.

All participating children underwent a series of ophthalmic tests to determine their visual, ocular, and refractive status. These included measures of best corrected visual acuity, tests of binocular and accommodative function, cycloplegic autorefraction (using the Shin-Nippon NVision-K 5001 open field autorefractor following instillation of 1.0% Tropicamide), ocular biometry (using the Lenstar LS900 instrument), and spectral domain OCT imaging. To ensure that choroidal thickness measures were not confounded by changes due to the effects of cycloplegic drugs on the choroid,^38^^,^^39^ OCT imaging was performed prior to cycloplegia. All testing was conducted during school hours between 9 AM and 3 PM.

OCT images of the right eye only were analyzed from those children with best corrected vision of 20/32 or better, no signs of strabismus, no history of (based on parental report) or evidence of ocular disease, and no history of (based on parental report) systemic disease that might affect the choroid or refractive status were included in the analyses. The 250 children included in the final analyses, comprised 100 Indigenous children and 150 non-Indigenous children (Table 1). These children had spherical equivalent cycloplegic refractive errors ranging from +6.57 to −1.00 diopters (D; mean +0.72 ± 0.70 D), and astigmatic refractive errors from 0.00 to −2.50 D (mean −0.48 ± 0.32 D). The Indigenous children had a significantly less hyperopic spherical equivalent refractive error compared to the non-Indigenous children (mean +0.52 ± 0.80 D versus +0.86 ± 0.58 D; *P* < 0.001). There were no other statistically significant differences between the Indigenous and non-Indigenous children in terms of age, gender balance, astigmatic refractive error, or axial length (all *P* > 0.05).

**Table 1.** Demographic and Visual Characteristics of the Indigenous and Non-Indigenous Children in the Study

	Indigenous Children (*n* = 100)	Non-Indigenous Children (*n* = 150)
Age, years	11.2 ± 3.4	12.0 ± 3.4
Percentage females	51%	50%
Spherical equivalent refraction, D	+0.52 ± 0.80	+0.86 ± 0.58
Cylindrical refraction, D	−0.49 ± 0.34	−0.47 ± 0.31
Axial length, mm	23.12 ± 0.73	23.13 ± 0.73
Best corrected vision, logMAR	−0.06 ± 0.08	−0.06 ± 0.07

All values presented are mean ± SD.

OCT imaging was performed using the Heidelberg Spectralis Instrument (Heidelberg Engineering, Heidelberg, Germany), which has previously been shown to provide highly repeatable measures of choroidal thickness in children.^16^ This device uses a super luminescent diode of central wavelength 870 nm for OCT imaging and provides cross sectional chorio-retinal images of digital axial resolution of 3.9 µm. Images were captured using enhanced depth imaging (EDI) to improve the visibility of the choroid,^4^ and each child had high-resolution OCT images collected from their right eye, including two repeated six-line “star” scans, each comprising 6, 20 degrees long foveal centered line scans with each line separated radially by 30 degrees. Frame averaging was used, with each OCT image being the average of 40 B-scans. Only scans with image quality index (QI) values > 20 dB were included for analysis (mean QI from all scans was 30.9 ± 3.1 dB).

### Analysis

Following image capture, all OCT scans were exported and analyzed using custom written software. Initially all OCT images were analyzed using automated algorithms based upon deep learning methods, to segment the outer boundary of the retinal pigment epithelium (RPE), and the inner boundary of the chorioscleral interface (CSI).^40^ The automated segmentation was then checked by an experienced masked observer who corrected any segmentation errors. The choroidal thickness across each scan, defined as the axial distance from the RPE to the CSI, was then calculated based upon the final corrected segmentation. The transverse scale of each child's OCT data was adjusted to account for ocular magnification using their individual ocular biometry and refraction data using previously described methods.^11^

The choroidal thickness data from each child were then analyzed to calculate the subfoveal choroidal thickness (choroidal thickness at the center of the fovea in each scan). The choroidal thickness across the central 5 mm diameter surrounding the fovea in each scan was analyzed to determine the average foveal (central 1 mm diameter surrounding the fovea), parafoveal (average thickness from 1 mm to 3 mm diameter), and perifoveal (average thickness from 3 mm to 5 mm diameter) choroidal thickness, over 8 different meridians (temporal, superior-temporal, superior, superior-nasal, nasal, inferior-nasal, inferior, or inferior-temporal). A small number of participants (*n* = 8; 5 Indigenous children and 3 non-Indigenous children) were unable to maintain stable fixation for sufficiently long to capture all six scan lines, and therefore their data were only included in the subfoveal analyses.

Repeatability of choroidal thickness measures was determined based upon analyses of the two repeated scans collected for each participant using the methods of Bland and Altman.^41^ The mean difference (95% limits of agreement) between the two repeated measures of choroidal thickness in the subfoveal, foveal, parafoveal, and perifoveal regions was +0.1 (−5.9 to + 5.7), −0.3 (−7.8 to +7.2), −0.5 (−10.2 to +9.2), and −0.4 (−8.5 to +7.7) µm, respectively, indicative of excellent repeatability.

All statistical analyses were carried out using IBM SPSS Statistics version 25. A univariate analysis of variance (ANOVA) was used to examine the effect of Indigenous status upon subfoveal choroidal thickness, including age and axial length as covariates. To assess the central 5 mm macular choroidal thickness, a repeated measures ANOVA was conducted with two within-subject factors (eccentricity [fovea, parafovea, and perifovea] and meridian [temporal, superior-temporal, superior, superior-nasal, nasal, inferior-nasal, inferior, or inferior-temporal]) and the between-subject factor of Indigenous status, with axial length and age included as covariates.

## Results

### Subfoveal Choroidal Thickness

**Figure 1.**
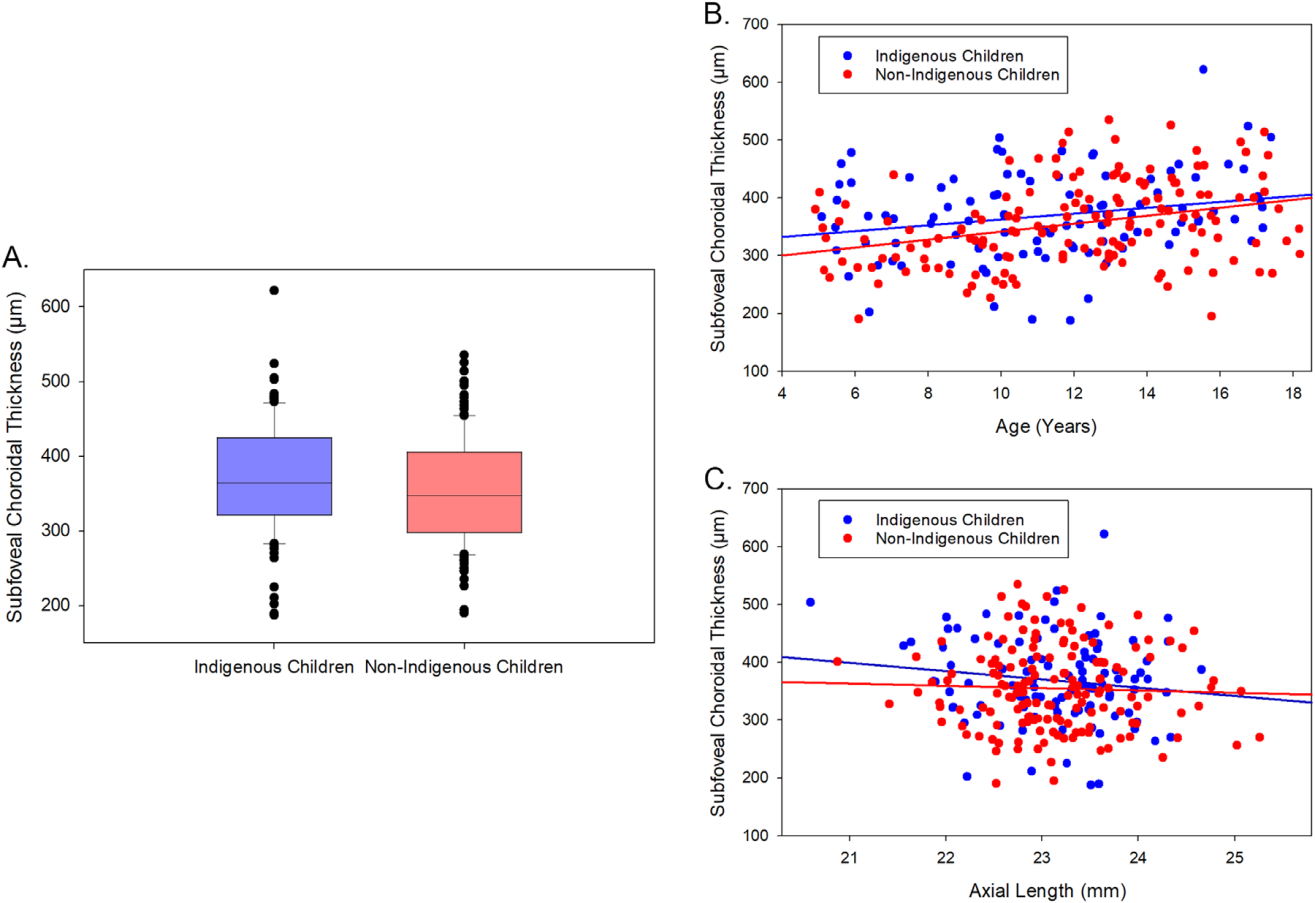
Subfoveal choroidal thickness in Indigenous (*n* = 100, *blue*) and non-Indigenous (*n* = 150, *red*) children (**A**). Relationship between age and subfoveal choroidal thickness (**B**) and axial length and subfoveal choroidal thickness (**C**) in this population of children is also illustrated (*blue and red solid lines* indicate best fit regression for the Indigenous and non-Indigenous children, respectively).

The mean subfoveal choroidal thickness of all children was 361 ± 74 µm (interquartile range = 308 to 409 µm) and was significantly thicker in Indigenous children (369 ± 75 µm, interquartile range = 321 to 425 µm) than non-Indigenous children (355 ± 73 µm, interquartile range = 298 to 406 µm; F_1,246_ = 4.29, *P* = 0.03; Fig. 1A). A significant positive association between subfoveal choroidal thickness and age (*β* = + 7.6 µm/year, r^2^ = 0.105, F_1,246_ = 30.15, *P* < 0.001; Fig. 1B) and a negative association between subfoveal choroidal thickness and axial length (*β* = −19.9 µm/mm, r^2^ = 0.030, F_1,246_ = 9.6, *P* = 0.002) was also found (Fig. 1C). No significant 2-way interactions between Indigenous status and age or axial length were observed (all *P* > 0.05). There was no significant effect of gender (*P* > 0.05).

### Macular Choroidal Thickness

**Figure 2.**
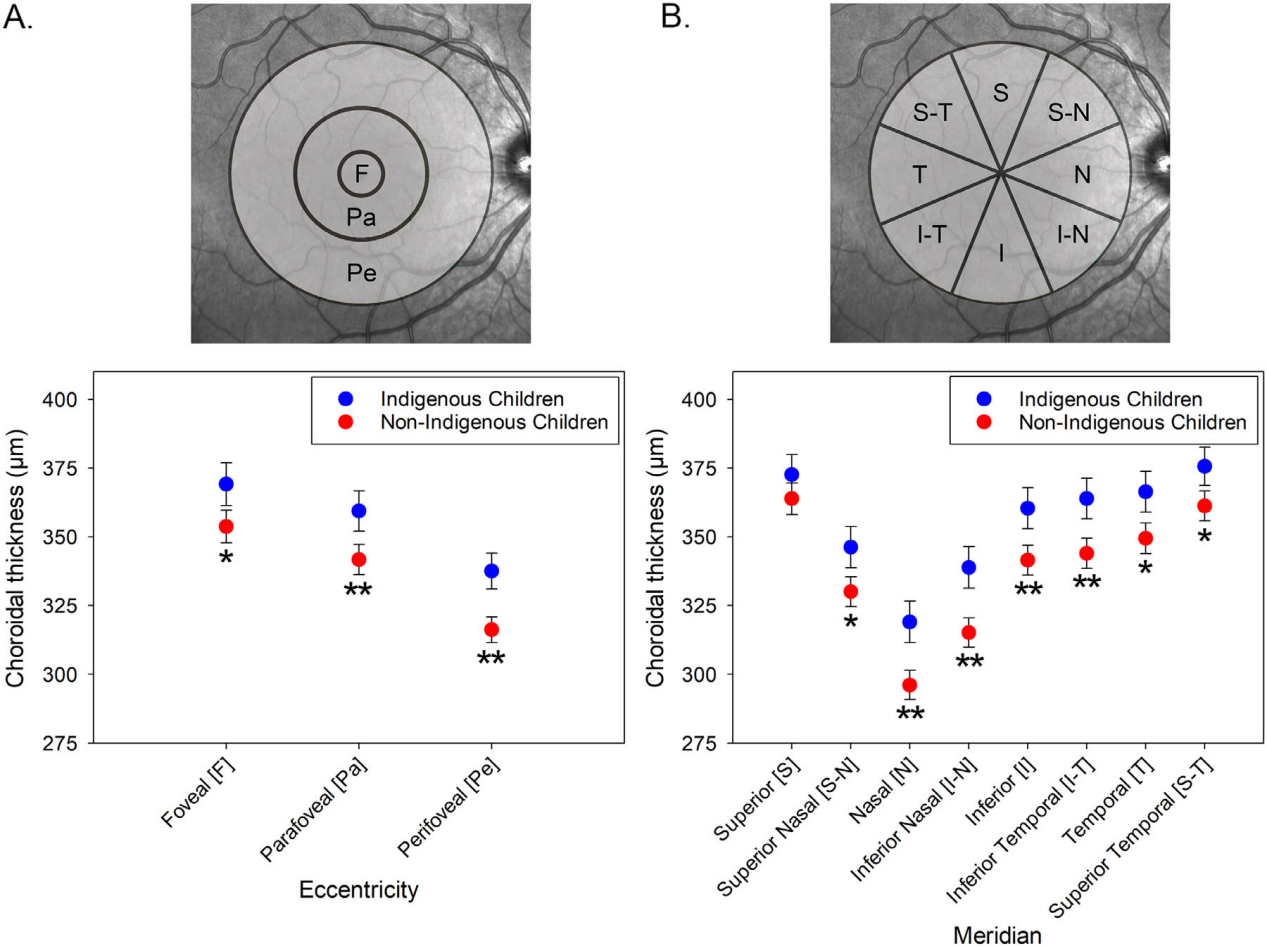
Variations in mean choroidal thickness in Indigenous and Non-Indigenous children over different macular eccentricities (**A**) and meridians (**B**). * *P* < 0.05 and ** *P* < 0.01 indicate a significant difference between Indigenous and non-Indigenous children. Error bars represent the standard error of the mean.

Across the central 5 mm, significant topographical variations in thickness were found in both Indigenous and non-Indigenous children (Fig. 2, Table 2). For both groups combined, the choroid was thickest in the central fovea, becoming significantly thinner in the parafovea (estimated mean difference 11 µm, 95% confidence interval [CI] = 9.2–12.6 µm) and perifovea (estimated mean difference 35 µm, 95% CI = 31–39 µm; all pairwise comparisons *P* < 0.001; Fig. 2A). The choroid was thinnest nasally and inferiorly and thickest superiorly and temporally (Fig. 2B). Similar to the subfoveal analyses, significant associations between macular choroidal thickness and age (F_1,238_ = 30.1, *P* < 0.001) and axial length (F_1,238_ = 7.1, *P* = 0.008) were also observed.

**Table 2.** Mean ± SD Choroidal Thickness (µm) From all Children With Complete Thickness Data Across the Central 5 mm Macular Region (*n* = 242)

		Eccentricity		
		Foveal	Parafoveal	Perifoveal
Meridian	Superior	364 ± 74	369 ± 71	369 ± 69
	Superior-nasal	360 ± 74	344 ± 71	306 ± 67
	Nasal	354 ± 74	318 ± 73	244 ± 66
	Inferior-nasal	355 ± 75	330 ± 73	288 ± 66
	Inferior	358 ± 74	349 ± 72	339 ± 66
	Inferior-temporal	361 ± 74	353 ± 73	342 ± 67
	Temporal	364 ± 74	360 ± 72	346 ± 69
	Superior-temporal	366 ± 73	370 ± 69	365 ± 65

**Figure 3.**
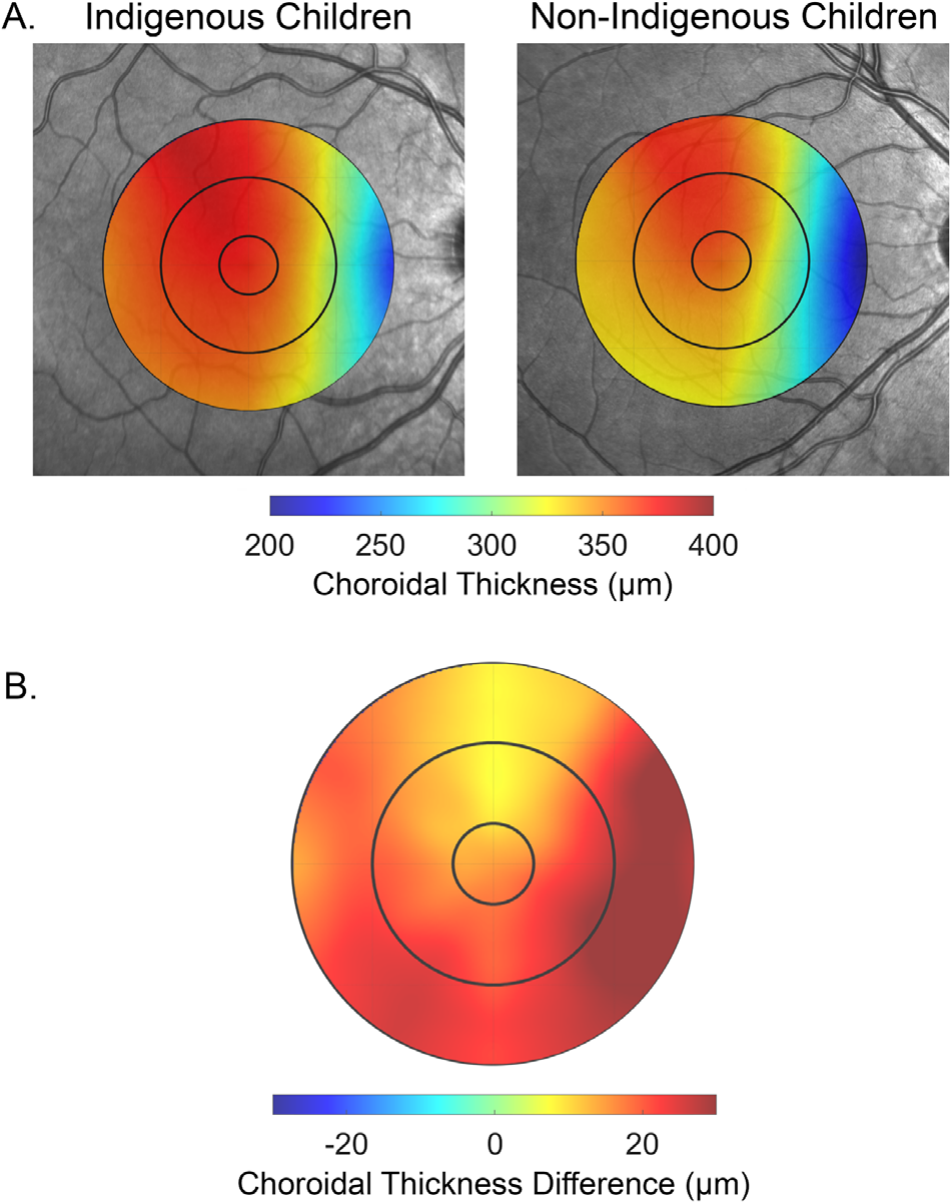
Topographical choroidal thickness maps across the central 5 mm macular region showing the mean choroidal thickness (**A**) for the Indigenous children (*left*) and the non-Indigenous children (*right*), and the mean difference in thickness between Indigenous and non-Indigenous children (**B**). Note that positive thickness difference values in **B** indicate a thicker choroid in Indigenous children.

The average macular choroidal thickness was significantly greater (F_1,238_ = 7.1, *P* = 0.008) in Indigenous children compared to non-Indigenous children (across all eccentricities and meridians, estimated mean difference = 22 µm, 95% CI = 6–39 µm). A significant interaction between meridian and Indigenous status was also observed (F_3.2,755_ = 4.0, *P* = 0.007), with the largest differences between Indigenous and non-Indigenous children observed in the nasal (estimated mean difference = 27 µm, 95% CI = 11–44 µm, *P* = 0.001) and inferior nasal (estimated mean difference = 28 µm, 95% CI = 12–45 µm, *P* = 0.001) meridians, and the smallest difference in the superior meridian (estimated mean difference = 14 µm, 95% CI = −4–31 µm, *P* > 0.05; Fig. 2B). However, there was no significant eccentricity by Indigenous status interaction (F_1.1,257_ = 1.7, *P* > 0.05) indicating that the difference in thickness between Indigenous and non-Indigenous children was similar between the foveal, parafoveal and perifoveal zones. Figure 3 illustrates the mean macular topographical thickness maps for the Indigenous children and non-Indigenous children, and the mean thickness difference between groups, highlighting the meridional differences in thickness between groups across the central 5 mm macular region.

## Discussion

This is the first study to examine the macular choroidal thickness of Indigenous Australian children, and establishes the normative ranges for this population. Compared to primarily Caucasian children living in the same rural geographic location (of similar age and axial length, and both exhibiting a low prevalence of myopia), Australian Indigenous children had a significantly thicker macular choroid. Previous reports of mean subfoveal choroidal thickness in populations of primarily non-myopic children from a variety of different countries,^8^^,^^9^^,^^11^^,^^13^^,^^15^^,^^30^^,^^42^ have ranged from 253 to 361 µm. In our population of mostly non-myopic children, the mean subfoveal thickness combined is at the upper level of these previous estimates in pediatric populations, and the mean thickness of Indigenous children is slightly higher than this again. Consistent with previous studies of healthy children, we observed a relatively wide range of choroidal thickness values in this population of healthy children (interquartile range of approximately 100 µm for subfoveal choroidal thickness). From a clinical perspective, these data suggest that when assessing choroidal thickness of Indigenous Australian children, a thicker than average subfoveal choroid is an expected normal finding, with the majority of the subfoveal thickness measures predicted to be between 321 and 425 µm.

In this primarily non-myopic population of children with a relatively wide range of ages (from 4 to 18 years), we found a significant increase in choroidal thickness associated with increasing age, with a 7.6 µm increase in subfoveal choroidal thickness per year observed during childhood development. These findings are consistent with previous cross-sectional^8^^,^^11^^,^^13^^,^^30^ and longitudinal^15^^,^^16^ studies of non-myopic children, that have estimated mean annual increases in choroidal thickness during childhood of between 4 and 16 µm per year. Also consistent with previous studies of children, choroidal thickness was negatively associated with axial length, however, the strength of this association for our population was weaker than that observed between choroidal thickness and age. This is most likely due to the relatively low prevalence of myopia and small spread of axial length in our population, because past studies, including higher proportions of myopic subjects (and, hence, a wider spread of axial length), have reported stronger associations between axial length and choroidal thickness in childhood.^9^^,^^14^

Across the entire cohort, significant topographic variations were also observed in choroidal thickness across the macular region, with central regions generally thicker than more peripheral regions, temporal regions thicker than nasal, and superior thicker than inferior. This topographical distribution of macular choroidal thickness is broadly similar to previous reports in children^11^^,^^30^^,^^43^ and adults.^5^^,^^44^ The difference in thickness between Indigenous and non-Indigenous children also showed significant meridional variations, with the most prominent differences in thickness observed in the nasal macula regions. Further research is needed to understand the underlying cause for these differences, however, given the highly vascular nature of the choroid, it is plausible that these differences in thickness may reflect differences in the density and distribution of choroidal blood vessels between Indigenous and non-Indigenous children. Differences in the magnitude and distribution of choroidal stromal components may also play a role.

Studies of normal eye growth^15^^,^^16^^,^^18^ and of myopia control interventions^45^^,^^46^ indicate that choroidal thickening is typically associated with slower eye growth, and, hence, a lower risk of myopia development and progression. The greater choroidal thickness observed in the Indigenous children may therefore be expected to be associated with slower eye growth and to therefore protect against the development of myopia in this population in the future. Although only a small number of studies have examined refractive error characteristics in Australian Indigenous populations, it is worth noting that Indigenous adults^37^ and children^36^ have generally been found to exhibit a lower prevalence of myopia compared to non-Indigenous populations. Previous studies of macula choroidal thickness in childhood have reported that the most prominent thinning of the choroid associated with myopia typically occurs between the fovea and the disc (i.e. nasal to the fovea).^11^^,^^43^ Given the association between myopia and choroidal thinning nasal to the fovea, we hypothesize that the greater choroidal thickness in nasal regions observed in Indigenous children may be a protective mechanism against the development of myopia later in life in Indigenous Australian populations. The meridional differences in choroidal thickness observed also suggest the potential for the presence of differences in peripheral eye shape associated with ethnicity, however, further research is needed to confirm this.

Our findings of a significantly increased choroidal thickness in Australian Indigenous children, may also provide some insights into the potential factors predisposing or protecting Australian Indigenous people from eye disease later in life. Studies indicate that Indigenous Australian adults exhibit a significantly higher rate of diabetic macular oedema compared to non-Indigenous Australians with diabetes.^47^ Conversely, Indigenous Australians have been reported to have a significantly reduced incidence of age-related macular degeneration (AMD) compared to non-Indigenous Australians, particularly for cases of intermediate and late AMD.^48^ Although a range of different genetic and environmental factors may underlie these previous findings in Indigenous adults, the documented positive association between choroidal thickness and diabetic macular oedema^24^ and negative association between choroidal thickness and progression of non-exudative AMD suggest that studies to examine choroidal thickness in Australian Indigenous adults and its association with macular disease, provides an important avenue of future research.

A limitation of this study is the cross-sectional study design, which limits the conclusions that can be drawn regarding the time course of variations in choroidal thickness occurring with age and the development of refractive error. Further longitudinal studies will help to better understand these changes and their relationship with eye growth. Although recruiting all participants living in the same rural location reduces the potential for environmental differences between groups to influence comparisons, it does limit the generalizations that can be made to children living in other locations. Further work exploring choroidal thickness of children living in urban as well as more remote regions will expand our understanding of choroidal thickness in Australian Indigenous children.

In conclusion, this study provides the first assessment of choroidal thickness in Australian Indigenous children and demonstrates a significantly greater choroidal thickness in this population. These findings help to establish the normative characteristics of the choroid in Indigenous Australians, which may assist in the future diagnosis and monitoring of choroidal abnormalities. Furthermore, although additional research is required to characterize the normal choroidal thickness of Indigenous Australians across a wider range of ages, our findings may have implications for the potential risk for the development of a range of conditions associated with choroidal changes later in life among Indigenous Australians.
